# Nurse-led, telephone-based follow-up after acute coronary syndrome yields improved risk factors after 36 months: the randomized controlled NAILED-ACS trial

**DOI:** 10.1038/s41598-021-97239-x

**Published:** 2021-09-06

**Authors:** Robin Henriksson, Daniel Huber, Thomas Mooe

**Affiliations:** Department of Public Health and Clinical Medicine, Umeå University, Östersund, Umeå, Sweden

**Keywords:** Cardiology, Risk factors

## Abstract

We investigated whether a nurse-led, telephone-based follow-up including medical titration was superior to usual care in improving blood pressure (BP) and low-density lipoprotein cholesterol (LDL-C) values 36 months after acute coronary syndrome (ACS). We screened all patients admitted with ACS at Östersund hospital, Sweden, between January 1, 2010, and December 31, 2014, for inclusion based on ability to participate in a telephone-based follow-up. Participants were randomly allocated to usual care or an intervention group that received counselling and medical titration to target BP < 140/< 90 mmHg and LDL-C < 2.5/< 1.8 mmol/L. The primary outcome was LDL-C at 36 months. Of 962 patients, 797 (83%) were available for analysis after 36 months. Compared to controls, the intervention group had a mean systolic BP (SBP) 4.1 mmHg lower (95% confidence interval [CI] 1.9–6.5), mean diastolic BP (DBP) 2.9 mmHg lower (95% CI 1.5–4.5), and mean LDL-C 0.28 mmol/L lower (95% CI 0.135–0.42). All *P* < 0.001. A significantly greater proportion of patients reached treatment targets with the intervention. After 36 months of follow-up, compared to usual care, the nurse-led, telephone-based intervention led to significantly lower SBP, DBP, and LDL-C and to a larger proportion of patients meeting target values.

**Trial registration**: ISRCTN registry. Trial number ISRCTN96595458. Retrospectively registered.

## Introduction

Despite considerable progress in management of cardiovascular disease (CVD), it remains the number one cause of death worldwide^1^. An increasing proportion of patients presenting with an acute coronary syndrome (ACS) survive, and secondary prevention is crucial to reducing further CVD complications. Regular physical activity, smoking cessation, and management of blood pressure and blood lipids are well-known and essential features of secondary prevention. However, patients often do not reach secondary preventive targets, as seen in the EUROASPIRE surveys and in number of studies^2–6^. Adherence to prescribed treatment is also insufficient^7^. Even though secondary prevention is a lifelong commitment, most studies lack long-term data on patient adherence and risk factor control. In a small French study that examined long-term adherence in a cohort of survivors of myocardial infarction, only 10% met all secondary preventive targets^8^. A number of strategies have been devised to improve secondary prevention, including different cardiac rehabilitation programs, smartphone applications, and telemedicine approaches. Telephone-based follow-up has shown some promise, but long-term data are lacking, and most studies have been small scale, essentially limiting external validity^9,10^.

The Nurse-based Age-independent Intervention to Limit Evolution of Disease after ACS (NAILED ACS) trial was an open randomized controlled trial carried out in the county of Jämtland, Sweden. The aim of the trial was to test the hypothesis that nurse-led, telephone-based follow-up and intervention, including physician-assisted medical titration, was superior to usual care as provided by the patient’s general practitioner (GP) in improving systolic blood pressure (SBP), diastolic blood pressure (DBP), and low density lipoprotein-cholesterol (LDL-C) after 36 months of follow-up. According to protocol, the primary outcome was mean LDL-C at 36 months. Details of the protocol and implementation have been previously published^11,12^. At one year of follow-up, an exploratory analysis that included LDL-C, SPB, and DBP showed a significant reduction in LDL-C and DBP and a non-significant trend toward lower SBP in the intervention group^13^. These are the final results of the NAILED ACS trial after 36 months of follow-up.

## Methods

### Trial design

The NAILED ACS-trial was an open, single-centre, prospective, randomised controlled intervention trial with two parallel groups. The aim of the trial was to examine whether a nurse-based telephone intervention was better than usual care in controlling the risk factors SBP, DBP and LDL-C, and in achieving a higher proportion of patients reaching set target levels of BP and LDL-C.

### Participants

The county of Jämtland, Sweden, has only one hospital (Östersund), which has a catchment area of around 130,000 people. During the inclusion period, which ran from January 1, 2010, until December 31, 2014, all patients admitted with ACS were eligible for inclusion and screened. The definition of ACS was either acute myocardial infarction type 1 (AMI: either ST-elevation myocardial infarction [STEMI] or non-STEMI [NSTEMI]) or unstable angina (UA)^14^. Exclusion criteria were based on the patient’s inability to participate in the telephone-based follow-up, and patients were excluded if they had hearing loss, aphasia, severe dementia, an inability to communicate in Swedish or English, or could not use a telephone. Participation in another ongoing trial was also considered a reason for exclusion. A previous study explored reasons for non-participation^12^.

### Randomisation

Randomisation occurred via computer allocation in blocks of four, with stratification based on type of ACS (AMI, UA) and sex. Patients were randomised into two parallel groups, a control group and an intervention group.

### Data collection and follow-up

During the initial hospitalisation, baseline data, demographic information, comorbidities, the use of medication and health status were collected via interview and medical records. At 1, 12, 24, and 36 months after hospital discharge, blood pressure (BP) readings and blood lipid measurements were collected via the closest health care provider. Shortly thereafter, a study nurse telephoned patients in both study groups.

**Figure 1:**
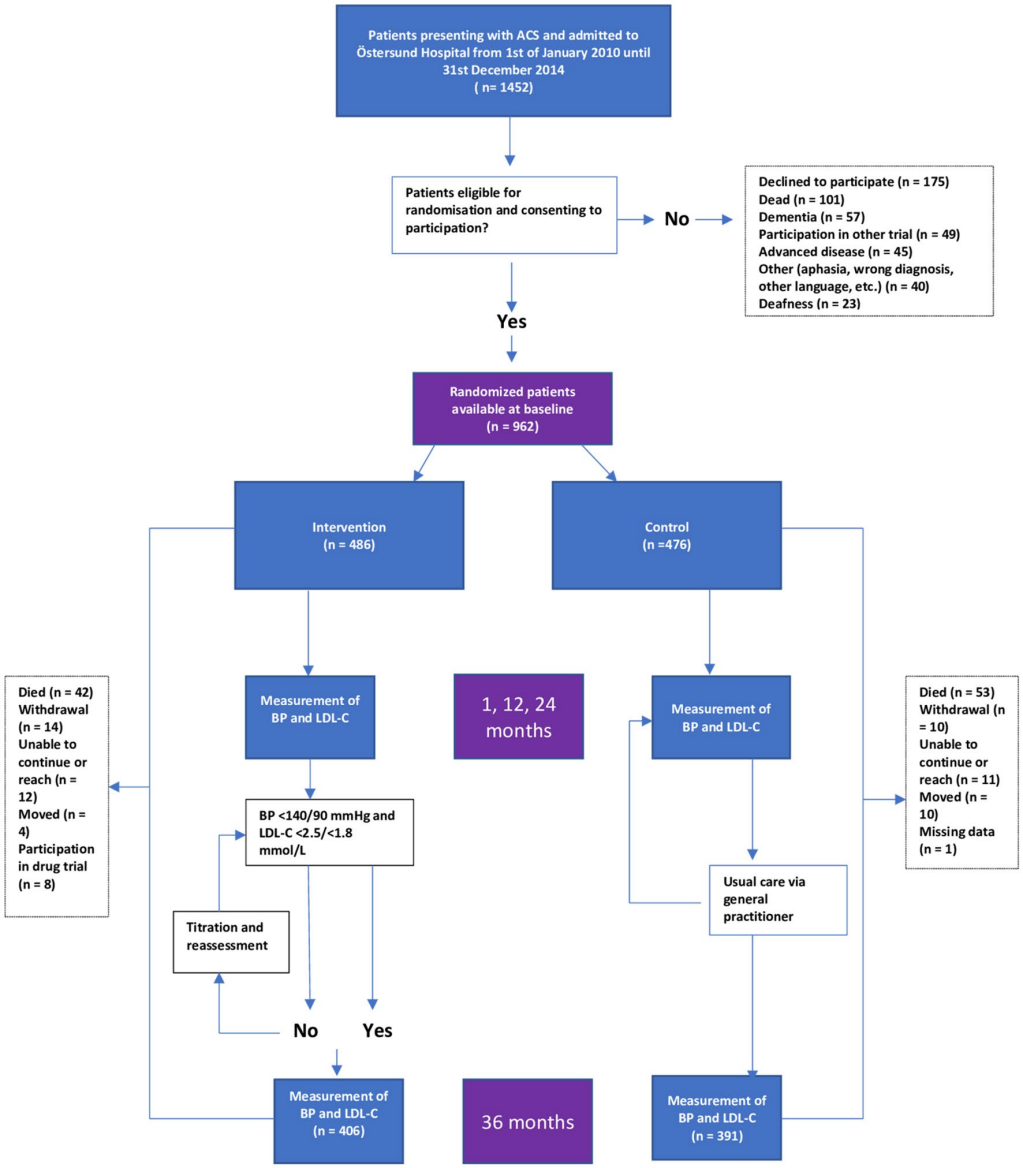
Study flow chart.

Patients in both the intervention and control group were interviewed in regard to general status, level of physical activity, smoking and medication intake. Results were recorded on pre-printed standardised forms and stored in binders organised by patient number. The data from standardised BP and LDL-C measurements were registered in both the electronic journal system and also on standardised paper forms. LDL-C values were calculated using Friedewald’s formula based on fasting values of cholesterol and triglycerides. Blood pressure readings were made with the patient in a seated position after 5 min of rest. Figure 1 show the study flow chart.

### Usual care

Regardless of whether randomised into the intervention or control group, all patients received the standard follow-up via the cardiology clinic. This standard follow-up include a visit to a cardiology nurse approximately one month after discharge, and after 2–3 months a visit to a cardiologist. This follow-up is the same for trial patients and non-trial patients. During the standard follow up via the clinic, patients receive general secondary preventive guidance in regard to lifestyle factors and an overview of medication and secondary preventive targets. In most cases, patients would then be referred to their GP for continued secondary preventive treatment. For both groups, secondary preventive medication was initiated in-hospital in accordance to national guidelines.

### Intervention group

During the telephone based follow-up patients randomised to the intervention were counselled on the importance of medical adherence, physical activity, and exercise and in applicable cases, smoking cessation. The study nurses were educated in regard to motivational interviewing. Patients in the intervention group were given advice regarding diet and exercise in accordance with recommendations by the National Food Administration and the National Board of Health and Welfare. Current smokers were given advice about smoking cessation and were recommended available resources. At follow-up, if an intervention patient had BP or LDL-C values above target levels, a physician would be contacted and the patient would have their medication titrated to achieve target levels. Titration was individualized and choice of medication and dosage was up to the treating study physician. Following every titration, a follow-up via telephone was scheduled approximately one month after titration, and if needed further medical adjustments was made.

### Control group

For the control group, there was no intervention or titration made by the study physicians and the patients received no counselling or medical advice from the study nurses. Patients simply provided BP and LDL-C measurements and were interviewed. The blood lipid and blood pressure measurements from the control group were available to both the study nurses and the patient’s primary care provider. The patients received what we refer to as usual care as previously described.

### Target levels

Target levels for BP and LDL-C were based on current local guidelines at the time of the trial. Target levels for BP were < 140/90 mmHg. In regard to LDL-C the initial target was < 2.5 mmol/L. In March 2013 there was a local guideline change for diabetic patients in which a lower target of < 1.8 mmol/L was set. The impact of this change has been previously published^15^. In 2017, local guidelines were updated again and a target LDL-C value of < 1.8 was set for all patients with a history of ACS.

### Statistical analysis

We performed analyses in accordance to an intention-to-treat principle, in which data were analysed regardless of patient adherence to treatment. We did not use imputation for missing data. Results are presented as means for continuous variables and as percentages for categorical variables. Baseline characteristics between the intervention and control groups were compared using t-tests for continuous variables and chi-square tests for categorical variables. Adjusted means were compared between the intervention and control groups via general linear regression, adjusted for our randomisation variables, sex, and type of ACS. Paired samples t-tests were used for comparisons within groups. A *P* < 0.05 was considered significant. All analyses were carried out using IBM SPSS v24.

### Power calculation

The power calculation was based on being able to detect a mean difference of 5 mmHg in SBP with a standard deviation (SD) of 19, and a mean difference of 0.5 mmol/L in LDL-C (SD 1.0). Based on a two-tailed alpha of 0.05 and 80% power, we calculated that a minimum of 200 participants in each study group would be needed. We included substantially more patients to maintain statistical power for analyses after long-term follow-up and in subgroups.

### Trial registration

The NAILED-ACS trial was registered in the International Standard Randomized Controlled Trial Number (ISRCTN) registry on August 24, 2011 (trial number: ISRCTN96595458). Unfortunately, before the strict requirement of prospective registration came to our attention, recruitment had already begun. Thus, this study is classified as retrospectively registered. We confirm that all related and on-going trials are now registered.

### Ethics

The Regional Ethics Committee, Umeå, approved the study on October 28, 2009. The study was conducted in accordance with relevant guidelines and regulations regarding scientific research. All participants signed an informed, written consent document (D-nr 09-142M).

## Results

### Participants

In total, 962 patients were randomised. Of 486 patients in the intervention group, 406 (83.5%) completed the 36-month follow-up. In the control group, there were 476 patients enrolled, and 391 (82.1%) completed the 36-month follow-up. In the intervention group, 42 patients died between randomisation and the 36-month follow-up, 14 chose to withdraw participation, 12 were unable to continue or were unreachable, 4 moved, and 8 participated in drug trials. In the control group, 53 patients died, 10 withdrew, 11 were unable to continue or were unreachable, 10 moved, and 1 patient had missing data (Fig. 1). There were no significant differences in baseline variables between the two groups (Table 1).

**Table 1:** Baseline characteristics of the randomised study population at 36 months.

	Control (n = 391)	Intervention (n = 406)	*P*
Mean age at inclusion, years (SD)	68.4 (10.9)	67.3 (10.7)	NS
Women (%)	29.7	27.6	NS
Smokers (current and ex) (%)	60.4	63.5	NS
**Qualifying event**			
UA (%)	11.3	8.6	NS
NSTEMI (%)	58.8	60.6	NS
STEMI (%)	29.9	30.8	NS
**Medical history**			
Diabetes (%)	17.9	18.7	NS
Atrial fibrillation (%)	10.2	12.1	NS
Heart failure (%)	0.8	1.7	NS
Myocardial infarction (%)	16.4	14.5	NS
Stroke or transient ischaemic attack (%)	5.6	6.2	NS
**Baseline measurements**			
SBP (mmHg) (SD)	132 (19)	131 (18)	NS
DBP (mmHg) (SD)	77 (11)	77 (10)	NS
LDL-C (SD)	2.18 (0.8)	2.18 (0.7)	NS

*NS* not significant, *SD* standard deviation.

### Blood pressure at the 36-month follow-up

The mean adjusted SBP was 133.5 (95% confidence interval [CI] 131.6–135.5) in the control group and 129.4 (95% CI 127.4–131.3) in the intervention group, for a difference in mean SBP of 4.1 mmHg (95% CI 1.9–6.5, *P* < 0.001). The mean adjusted DBP was 78.6 mmHg (95% CI 77.3–79.9) in the control group and 75.7 mmHg (95% CI 74.4–76.9) in the intervention group, resulting in a difference in mean DBP of 2.9 mmHg (95% CI 1.5–4.5, *P* < 0.001).

### LDL-C at the 36-month follow-up

The mean adjusted LDL-C was 2.42 mmol/L (95% CI 2.3–2.54) in the control group and 2.14 mmol/L (95% CI 2.01–2.26) in the intervention group. The resulting difference between groups in LDL-C was 0.28 mmol/L (95% CI 0.135–0.42, *P* < 0.001).

### Changes between 1 and 36 months

The comparisons of SBP, DBP, and LDL-C between 1 month and the 36-month follow-up within the two groups showed that in the control group, mean SBP increased non-significantly by 1.2 mmHg (95% CI − 0.9 to 3.3), mean DBP increased non-significantly by 1.2 mmHg (95% CI − 0.04 to 2.4), and mean LDL-C rose by 0.21 mmol/L (95% CI 0.1–0.31). In the intervention group, mean SBP decreased by 2.2 (95% CI − 0.3 to − 4.2), mean DBP decreased non-significantly by 1.1 mmHg (95% CI − 0.04 to 2.3), while mean LDL-C was 0.017 mmol/L non-significantly lower (95% CI − 0.08 to 0.11).

### Treatment targets

**Figure 2:**
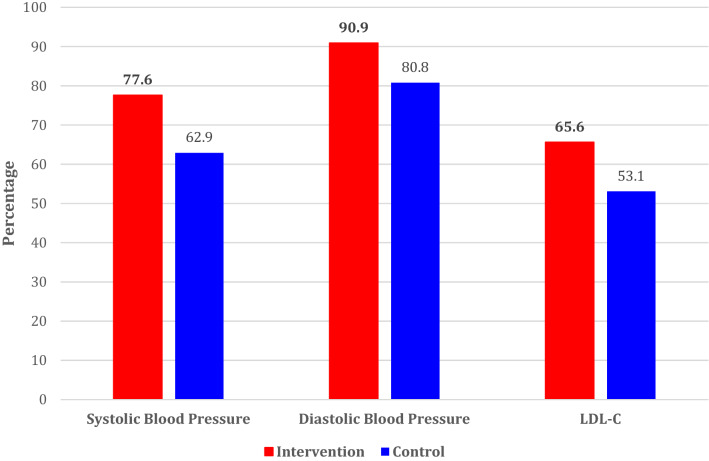
The proportion of patients reaching treatment targets at 36 months of follow-up.

Figure 2 depicts the proportion of patients reaching treatment targets at 36 months of follow-up. The proportion of patients within target levels for SBP was 62.9% in the control group, compared to 77.6% in the intervention group (*P* < 0.001). This proportion for DBP was 80.8% of patients in the control group reaching the target vs 90.9% in the intervention group (*P* < 0.001). For LDL-C targets, 53.1% in the control group met targets vs 65.6% in the intervention group (*P* < 0.001).

### Use of medicine at the 36-month follow-up

Table 2 shows the use of medicine at the 36-month follow-up. In the intervention group, the use of calcium channel blockers was more common, and the use of statins, angiotensin-receptor blockers, and anticoagulants as well as diuretics were numerically higher, although non-significantly compared to the control group.

**Table 2:** Medication at 36 months.

	Control (n = 391), %	Intervention (n = 406), %	*P*
Statins	86.9	90	0.16
Diuretics	32.2	38.7	0.06
Beta-blockers	88.7	86.6	0.38
Angiotensin-converting enzyme inhibitors	47.4	44.4	0.39
Angiotensin receptor blockers	29.4	35.6	0.06
Calcium channel blockers	28.6	35.7	0.03
Aspirin	83.5	78.9	0.1
Other antiplatelet drug^a^	8	8.2	0.92
Anticoagulants	9.8	13.9	0.07

^a^Other antiplatelet drug, e.g., clopidogrel, ticagrelor.

### Trends over time

**Figure 3:**
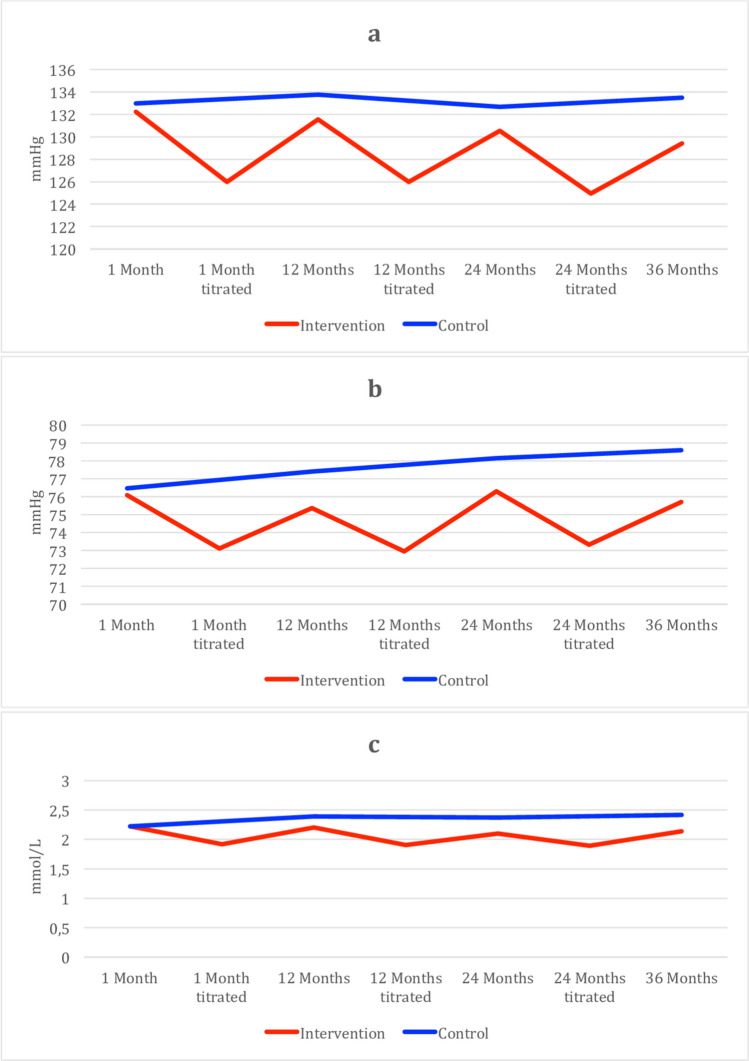
Adjusted mean values for SBP (**a**), DBP (**b**), and LDL-C (**c**) at assessments at 1, 12, 24, and 36 months and after titration. *SBP* systolic blood pressure, *DBP* diastolic blood pressure, *LDL-C* low-density lipoprotein cholesterol. Means adjusted for type of ACS and sex.

As seen in Fig. 3 and Table 3 below, the adjusted means for SBP, DBP, and LDL-C showed a clear difference between groups. For the intervention group, the titrations were associated with a distinct decrease in SBP, DBP, and LDL-C, although at the subsequent scheduled annual assessment, the effect of the intervention had decreased.

**Table 3:** Adjusted means over time.

	Control	Intervention
**Systolic blood pressure**		
1 month	133	132.2
1 month titrated	N/A	126
12 months	133.8	131.6
12 months titrated	N/A	126
24 months	132.7	130.5
24 months titrated	N/A	124.9
36 months	133.5	129.4
**Diastolic blood pressure**		
1 month	76.5	76.1
1 month titrated	N/A	73.1
12 months	77.4	75.4
12 months titrated	N/A	73
24 months	78.2	76.3
24 months titrated	N/A	73.3
36 months	78.6	75.7
**LDL-C**		
1 month	2.22	2.22
1 month titrated	N/A	1.92
12 months	2.39	2.2
12 months titrated	N/A	1.9
24 months	2.38	2.1
24 months titrated	N/A	1.89
36 months	2.42	2.14

## Discussion

In this randomised controlled trial, a nurse-led telephone-based follow-up that included medical titration was superior to usual care. After 36 months of follow-up, the intervention group had significantly lower values for SBP, DBP, and LDL-C. The proportions of patients reaching target values were also significantly higher in the intervention group. There was a trend towards rising values in the control group, and lower values in the intervention group. The differences were numerical, and not statistically significant with the exception of lower SBP in the intervention group and higher LDL-C in the control group. Overall, this result suggests that the intervention promoted achievement of lower levels and helped patients avoid the rise, particularly in LDL-C, seen in the control group.

### Effect of the intervention on BP and LDL-C and clinical relevance

At the end of the study follow-up at 36 months, comparing the intervention group to controls, the mean SBP was 4.1 mmHg lower, mean DBP was 2.9 mmHg lower, and mean LDL-C was 0.28 mmol/L lower. A lowering of BP and LDL-C in secondary prevention reduce cardiovascular events as shown in several prior studies.

The clinical relevance of the present numerically small differences between the intervention and control group can be placed in the context of previously published analyses, such as results of a meta-analysis showing that a reduced SBP by 10 mmHg translated into a 22% reduction in coronary heart disease^16^. Regarding LDL-C, other studies have shown that a reduction in LDL-C by 1 mmol/L resulted in a decreased relative risk of cardiovascular death by approximately 20%^17–19^. The combined effect of lowering blood pressure and LDL-C and the effect of the marked reduction in risk factor levels after titration is difficult to estimate.

Effects on mortality and morbidity were not the focus of this study. A numerically higher number of participants had died by the 36-month follow-up in the control group as compared to the intervention group (53 vs 42) corresponding to percentages of 11% and 8.6%, respectively, from randomisation to the 36-month follow-up. A separate study regarding adjudicated clinical endpoints in the entire NAILED trial population is recently completed and will be reported in accordance with the study plan (ISRCTN30433343; Scientific Reports 2021, accepted for publication).

### Target level achievement

Secondary preventive measures following acute coronary syndrome have room for improvement. The proportion of patients reaching target values for blood pressure and LDL-C tends to be inadequate, as was evident in the large Euroaspire IV-survey^5^, in which roughly two-fifths of patients reached treatment targets for blood pressure (140/90) and less than two-thirds attained treatment targets for LDL-C (< 2.5 mmol/L). In the present study, a comparatively larger proportion of patients in the intervention group reached treatment target levels (77.6% for SBP, 90.9% for DBP, and 65.5% for LDL-C). Compared to the results in the Euroaspire IV survey, our control group exhibited a higher proportion of patients who achieved treatment target levels at 36 months of follow-up for blood pressure (62.9% for SBP and 80.8% for DBP). The proportion of patients reaching target levels for LDL-C were in line with the results of the survey, with 53.1% reaching target levels. The higher proportion of patients in the control group who achieved treatment target levels compared to patients in the Euroaspire IV-study needs to be considered when evaluating the effect of the intervention. The relative improvement in the intervention group was achieved despite a well-treated control group.

### Intervention and titration

Our results demonstrate that although the intervention did not result in a continuous reduction of BP and LDL-C a reduced risk factor burden was maintained throughout the study period. Shortly after titration, the difference was more pronounced, but the effect abated, as can be seen in Fig. 3. Even though the effect was not lasting, the reduction in overall risk factor burden in the intervention group, measured as a reduced area under the curve, may be speculated to add to the benefit related to the lower point estimates of blood pressure and LDL-C. Of note, the titration could result in long lasting lowering of BP and LDL-C on an individual basis, but on a group level the effect diminished over time. We can only speculate as to why the effect of the titration declined over time for the whole group. Some patients that were previously within target levels could experience a worsening of BP and LDL-C over time which could be due to a number of factors, such as poor medical adherence, changes in lifestyle factors or for other medical reasons.

### Follow-up and medication

At the yearly follow-up, patients were interviewed and asked about their current medication. Study nurses also evaluated both prescription and laboratory data to identify discrepancies such as reporting statin adherence but having increased LDL-C. At the 36-month follow-up, there were no significant differences regarding use of medication apart from a higher proportion of intervention patients receiving calcium channel blockers, although there was a nonsignificant trend towards higher use of statins, diuretics, and angiotensin receptor blockers. However we do not have data about the dose of the different blood pressure lowering medications but it is possible that the intervention group in general were prescribed higher doses. This is supported by our previous findings that the intervention led to increased adherence to statins and a greater use of high-intensity lipid lowering therapy^20^.

Overall, most patients were treated with medications in accordance with European Society of Cardiology guideline recommendations^21,22^.

This indicates that a reported high proportion of patients on treatment does not automatically translate to a high proportion reaching treatment target levels. This could be due to a number of reasons, such as inadequate doses or poor adherence.

Poor adherence to secondary preventive medications has been previously described^23,24^. It is possible that the yearly instructions on blood pressure measurement and blood lipid testing led to a higher proportion of patients with adequate treatment in both groups, as compared to numbers seen in the previous referenced studies. We earlier examined adherence to statin treatment specifically in NAILED ACS study participants with a mean of 3.9 years of follow-up. In the intervention group, 89% were adherent compared to 85% in the control group, so figures were high in both groups^20^.

A Norwegian study on adherence to secondary preventive drugs after myocardial infarction with up to 2 years of follow-up showed higher adherence compared to older studies, but still slightly lower than in the present study^25^.

Another explanation for inadequate secondary prevention is therapeutic or clinical inertia which has long been acknowledged and is defined as not acting in accordance with or adhering to guidelines in the treatment of various symptoms and diseases^26,27^. This inertia is likely part but not solely the explanation for the higher mean BP and increased LDL-C levels in the control group. The goal-oriented medical titration in the intervention group may have helped to lower this therapeutic inertia and aid in achieving treatment targets. Titration was not always possible, either because of non-adherence by the patient or decision of the study physician because of an already maximum dosage, co-morbidity, or adverse effects.

### Relevance of NAILED ACS

Comparing our results to other trials are difficult because of a lack of long-term perspective^9,10,28^. Other nurse-led or nurse-coordinated secondary prevention studies have also shown promising results but direct comparisons with the NAILED ACS trial are difficult to make due to heterogeneity in trial participants, design and time frame^29–32^.

Secondary prevention should be seen as a lifelong engagement, and this study shows that a nurse-led telephone-based intervention can improve control of relevant risk factors in the long-term. The overall design of the NAILED-ACS trial could be integrated into clinical practice, at least for developed nations, in a similar setting to diabetic follow-up in primary care. If so, further research with a focus on clinical outcomes is warranted.

### Strengths and limitations

The patients included in this study consist of a representative and clinically relevant population. The Jämtland county in middle Sweden consist of both urban and rural settings. There is only one hospital with one cardiology clinic, which enabled us to conduct the study in a controlled manner; however, the single centre design might limit external validity. The trial population in NAILED ACS consist of relatively unselected ACS patients and represents patients typically encountered in a clinical setting. The population and setting is comparable to other western countries. To our knowledge, no other population-based long-term secondary preventive intervention studies have focused on telephone-based follow-up. One limitation of this study is that the control patients received instruction each year to measure their blood pressure and blood lipid levels and had a short interview over the telephone with the study nurses. This interaction could remind the patient of the importance of blood pressure management and medication adherence. An underestimate of the effect of the intervention is therefore possible. An analysis of the importance of lifestyle factors was beyond the scope of the present trial.

## Conclusion

After 36 months of follow-up, the nurse-led, telephone-based intervention led to significantly lower SBP, DBP, and LDL-C values and increased the proportion of patients reaching their targets. Our data imply that a secondary prevention strategy must be sustained beyond the first year to maintain risk factor reduction.

## Data Availability

Data are available upon request to the corresponding author. Data cannot be made publicly available in a repository because of legal regulations.
